# Immunoaffinity-enriched salivary small extracellular vesicles in periodontitis

**DOI:** 10.20517/evcna.2023.48

**Published:** 2023-12-30

**Authors:** Chun Liu, Chaminda Jayampath Seneviratne, Carlos Palma, Greg Rice, Carlos Salomon, Ramin Khanabdali, Sašo Ivanovski, Pingping Han

**Affiliations:** ^1^Epigenetics nanodiagnostic and therapeutic group, Center for Oral-facial Regeneration, Rehabilitation and Reconstruction (COR3), School of Dentistry, The University of Queensland, Brisbane, QLD 4006, Australia.; ^2^INOVIQ Limited, Notting Hill, VIC 3168, Australia.; ^3^Translational Extracellular Vesicles in Obstetrics and Gynae-Oncology Group, University of Queensland Centre for Clinical Research, Faculty of Medicine, Royal Brisbane and Women’s Hospital, The University of Queensland, Brisbane, QLD 4029, Australia.

**Keywords:** Immunoaffinity capture, salivary extracellular vesicles, periodontitis diagnosis

## Abstract

**Aim:**

aliva extracellular vesicles (EVs) serve as a significant reservoir of biomarkers that may be of clinical use in disease diagnosis. Saliva, however, contains EVs of both host- and bacterial- origin. Identifying suitable EVs for disease diagnosis involves enriching host EVs and limiting non-host contamination with effective isolation methods. The objectives of this research were: (1) to evaluate the salivary EVs enrichment in 12 periodontally healthy patients by two different methods: size exclusion chromatography (SEC) and bead-based immunoaffinity capture (EXO-NET^®^); (2) to analyze the variance expression of inflammatory cytokines in EXO-NET-enriched EVs, comparing individuals with periodontitis (*n* = 20) to non-periodontitis (*n* = 12).

**Methods:**

Whole unstimulated saliva samples were collected from 12 periodontally healthy and 20 periodontitis patients. EVs were isolated from the 12 non-periodontitis patients using SEC (referred to as SEC-EVs) and EXO-NET (referred to as EXO-NET EVs), after which their total protein content, 37 EV surface markers, and bacterial pathogens expression were compared. Subsequently, the inflammatory cytokines expression levels (interleukin-IL-6, IL-1β, IL-8, and IL-10) in EXO-NET EVs were measured for non-periodontitis and periodontitis.

**Results:**

EXO-NET EVs contained more EV-specific protein and substantially higher expression of EV surface markers (CD9, CD81, CD63), but less pathogenic DNA was detected compared to that in SEC-EVs. Additionally, EXO-NET EVs from periodontitis patients contained higher amounts of IL-6 and IL-8, and decreased IL-10, compared to those from non-periodontitis patients.

**Conclusion:**

The findings suggest that immunoaffinity capture (EXO-NET) is a dependable method for salivary EVs enrichment, resulting in a higher yield of host EVs with reduced bacterial DNA detection compared to SEC. Furthermore, the research proposes that immunoaffinity capture enriched EVs can function as biomarkers for periodontitis, demonstrated by an increased expression of proinflammatory cytokines from periodontitis patients.

## INTRODUCTION

Periodontitis is a highly prevalent, chronic oral disease characterized by an imbalance between microbiota and the host's inflammatory response^[1-3]^. In periodontitis patients, an abnormal composition of pathogenic bacteria (under the gingiva) triggers an exaggerated immune response, resulting in chronic inflammation, causing damage and destruction to the supportive periodontal tissues surrounding the tooth, such as gingiva and periodontal ligament^[1-3]^. Furthermore, chronic periodontitis is associated with systemic diseases, such as diabetes^[4]^, hemochromatosis^[5]^, cardiac disease, and complications of pregnancy^[6-8]^. The development of periodontitis is attributed to the accumulation of dental plaque that leads to inflammation in the periodontium and subsequent loss of periodontal tissue^[2]^. This, in turn, leads to increased inflammation in both local (periodontium) and systemic (saliva and serum) regions^[3]^. If left untreated, it can progress to a severe form of periodontitis, resulting in tooth loss; therefore, timely diagnosis is paramount for decreasing patient morbidity^[9]^. The existing diagnostic methods primarily assess disease activity based on a previously established clinical tissue loss and involve techniques such as x-ray radiography to identify alveolar bone loss or manual probing to measure periodontal pocket depth (PPD) and detect bleeding on probing (BOP)^[1-3,10]^. These techniques are not capable of detecting the current disease activity. Therefore, identifying real-time molecular biomarkers for periodontitis represents a significant and informative alternative adjunct diagnostic tool.

Saliva, for example, has the potential to be a powerful non-invasive diagnostic tool that provides insights into both local and systemic health, as it contains a variety of biological molecules^[11]^, including proteins, mRNA^[12]^, EVs, anti-COVID-19 antibodies^[13,14]^, microbiota, among others^[15]^. Whole saliva contains a heterogeneous mixture of soluble analytes, which are found at relatively low concentrations and are prone to degradation within the oral cavity^[15]^. This susceptibility can potentially limit their effectiveness as diagnostic biomarkers. However, analytes associated with EVs are protected against degradation, making them a more reliable, non-invasive option for liquid biops^[16]^. Salivary EVs liquid biopsy may represent a simple and patient-acceptable method for frequent monitoring and early detection of periodontitis^[17-20]^. Additionally, salivary EVs may contain specific biomolecules (proteins, nucleic acids, and lipids) that are relevant for disease progression, which may enhance the detection sensitivity and reduce the impact of the complicated saliva environment^[16,21]^. Current research has demonstrated that the components of salivary EVs are superior diagnostics markers for periodontitis compared to whole saliva^[22-28]^. However, the absence of standardized methods for isolating and enriching EVs has constrained research in this area. Therefore, continuous efforts to develop and refine EV enrichment techniques are essential to propel advancements in the field.

In a recent review^[19]^, various methods have been employed for salivary EV enrichment, such as ultracentrifugation (UC), size exclusion chromatography (SEC), and chemical precipitation, each with unique advantages and limitations. UC has often been used to enrich particles of similar sedimentation or buoyant density; however, it is time-consuming and may lead to protein or EV aggregates^[29-31]^. SEC is a chromatographic technique that separates particles based on their hydrodynamic size, including membranous and non-membranous extracellular particles^[29]^. Chemical precipitation is a cost-effective option but may result in low yield and co-precipitation of contaminants^[29]^. It is worth noting that UC, SEC, and chemical precipitation methods separate extracellular particles based on physicochemical properties and are unable to distinguish between the host-derived and microbiota-sourced EVs. In contrast, the bead-based immunoaffinity approach utilizes antibodies specific to the host that are coated onto beads, enabling the selective targeting and capture of host-derived EVs with particular surface markers, such as CD markers. This method has the potential to exclusively enrich host-derived EVs through these targeted CD surface markers, thereby improving the specificity and ensuring the isolation is limited to the desired host EVs. Such advancements in isolation techniques bolster the reliability of research detecting host-response mechanisms and mitigate concerns related to microbial EV interference^[32]^. Furthermore, there is limited data characterizing the use of bead-based immunoaffinity for isolating salivary EVs in periodontitis patients.

In addition to EV enrichment methods, there is an evolving interest in defining the molecular contents that are carried by the vesicles, known as cargo, and their role in periodontitis pathogenesis. Several studies have investigated the cargo of salivary EVs in the field of periodontology, particularly components of protein^[22]^, cytokines^[33,34]^, mRNA^[23,35]^, microRNAs^[24,25]^, DNA methylation^[26,27]^, and EV surface markers (e.g., CD9 and CD81)^[28]^. In particular, cytokines associated with EVs may contribute to the modulation of immune and inflammatory processes within recipient cells^[36]^. The presence of cytokines in salivary EVs could indicate an ongoing immune response to oral pathogens, helping to regulate the inflammatory response to microbial invasion. Currently, there is limited literature on the exploration of cytokine profiles within salivary EVs during the pathogenesis of periodontitis. As key inflammation biomarkers, it is well-documented that periodontitis is linked to increased levels of proinflammatory cytokines [IL-6, tumour necrosis factor alpha-TNF-α, matrix metalloproteinase-8 (MMP-8), IL-1β]^[37]^ and reduced levels of anti-inflammatory cytokines (IL-8 and IL-10) in saliva and periodontal tissues^[38-42]^. Nonetheless, the variability of salivary cytokines among individuals restricts its utility in diagnosing periodontal disease. Previously, we have demonstrated that salivary EVs exhibited more robust and enriched profiles compared to whole saliva samples^[25,27,35]^. Therefore, it becomes important to understand periodontitis disease progression by exploring the cytokine profiles of salivary EVs in both periodontally healthy (non-periodontitis) and periodontitis patients. This study aims to use non-periodontitis saliva as a sample source to identify the optimal technique for enriching salivary EVs. Subsequently, it investigates cytokines within salivary EVs as potential biomarkers for the diagnosis of periodontitis.

## METHODS

**Figure 1:**
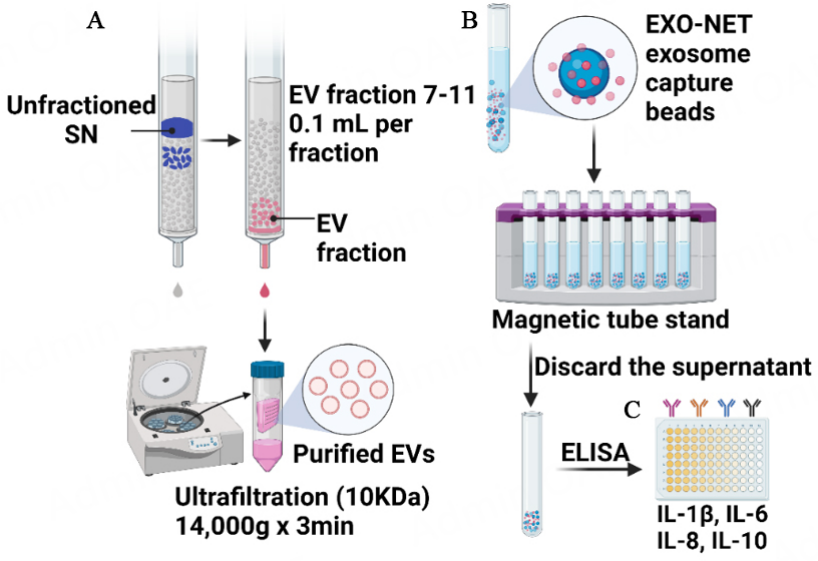
An experimental workflow for the isolation of EVs and the use of ELISA to analyze cytokines levels of EXO-NET EVs. (A) Size exclusion chromatography for EV isolation, referring to SEC-EVs in the following text; (B) Bead-based immunoaffinity capture (EXO-NET), referring to EXO-NET EVs in the following text; (C) ELISA for IL-6, IL-8, IL-10, and IL-1β for EXO-NET EVs from 12 non-periodontitis and 20 periodontitis patients. SN: Supernatant.

As illustrated in Figure 1, the study utilized two different methods to isolate EVs from non-periodontitis saliva samples (*n* = 12): SEC and bead-based immunoaffinity capture (EXO-NET). Subsequently, EXO-NET beads were employed to isolate EVs from periodontitis (*n* = 20) and non-periodontitis (*n* = 12), and enzyme-linked immunosorbent assay (ELISA) was conducted for the revealing of IL-6, IL-8, IL-10, and IL-1β cytokines.

### Patient recruitment and saliva collection

Approval for the study was granted by the Human Research Ethics Committee at The University of Queensland (HREC No. 2023000467) and the Metro North Hospital and Health Service (approval No. 54584). The study enrolled a total of 32 participants with written consent, comprising 12 non-periodontitis and 20 stages III/IV periodontitis cases as previously described^[12]^, following the latest 2017 periodontitis stage and grading guidelines^[43]^. The inclusion criteria for patient selection were as follows:

● The non-periodontitis control group comprises individuals aged 18 years or older with no history of periodontitis and PPD (Probing Pocket Depth refers to the measurement of the depth of the gingival sulcus or periodontal pocket) ≤ 3 mm.

● According to the latest periodontitis stage guidelines^[43]^, periodontitis has four different stages: early periodontitis (Stage I), moderate periodontitis (Stage II), severe periodontitis (Stage III), and severe with tooth loss periodontitis (Stage IV). We recruited Stages III and IV periodontitis in our study. Stage III and IV periodontitis are aged 18 years or older participants having ≥ 30% of sites with PPD ≥ 3 mm, CAL (Clinical Attachment Loss is the sum of the probing pocket depth and the distance from the base of the pocket to the point where the periodontal fibers attach to the tooth) ≥ 5 mm, and at least five sites with PPD ≥ 5 mm on at least three non-adjacent teeth, with radiographic evidence of bone loss extending from the root of the tooth by one-third or more.

All patients with no self-reported smoking history, no uncontrolled diabetes history, no xerostomia, and no long-term use of immunosuppressive or anti-inflammatory drugs were included.

Participants were instructed to fast and refrain from drinking and eating for at least one hour before saliva collection (from 8.00 am to 12.00 pm). Subsequently, participants rinsed their mouths with approximately 10 mL of water to eliminate any food debris. Saliva samples were collected through expectoration of unstimulated whole saliva (~ 5 mL) and were immediately preserved at -80 °C at the School of Dentistry and Oral Health Center at the University of Queensland.

### Salivary EVs enrichment by SEC and EXO-NET

SEC columns were used to isolate EVs based on particle size. The non-periodontitis salivary EVs were enriched using SEC columns (miniPURE-EVs, HansaBioMed, Lonza, QLD, Australia) adhering to the manufacturer’s protocol [Figure 1A] and as detailed in prior descriptions^[26,27]^. Saliva was diluted in a ratio of 1:1 of Dulbecco’s Phosphate Buffered Saline (DPBS, 1×, without calcium, magnesium, and Phenol Red; In Vitro Technologies Pty Ltd), then 300 μL saliva was transferred to centrifugation to remove debris, apoptotic bodies and microvesicles at 300 *g* for 15 min, 1,600 *g* for 15 min, 16,000 *g* for 20 min at 4 °C. Fractions 7 to 11 (100 μL each) were collected after the loading the supernatant to the SEC column and concentrated to 100 μL utilizing an Amicon Ultra 0.5 Centrifugal Filter Unit (10 kDa, Merck Millipore, QLD, Australia) *via* centrifugation at 4,000 *g* at 4 °C. SEC-EVs were used for the subsequent sections.

The bead-based immunoaffinity capture method [Figure 1B] utilizes microbeads that are coated with antibodies specific to surface markers present on the host EVs. EVs were captured following the manufacturer’s protocol. The beads (EXO-NET®, INOVIQ Ltd, Australia) and sample were equilibrated to room temperature. An aliquot of the 300 μL sample was mixed with the 28 μL beads by pipetting, and the mixture was flicked to ensure proper mixing. After incubating the mixture on a non-magnetic tube holder for 15 min, the tube was placed on a magnetic tube stand until the supernatant became clear. The supernatant was meticulously aspirated, and the bead pellet underwent three successive washes with PBS. It is noted that no serial centrifuge, such as at 300*g*, 2,600*g*, and 16,000*g*, was performed to remove cell debris and large particles before incubating with immunoaffinity microbeads (EXO-NET®, INOVIQ Ltd, Australia). EVs captured by the microbeads are referred to as “EXO-NET EVs”.

### Salivary SEC-EVs characterization

SEC-EVs were characterized using transmission electron microscopy (TEM), Nanoparticle tracking analysis (NTA), and EV purity as previously described^[26,27]^ and followed the guideline from Minimal information for studies of extracellular vesicles 2018 (MISEV2018)^[44]^.

TEM was used to explore EV morphology. The SEC-EVs samples were fixed in 4% paraformaldehyde and adsorbed onto Formvar carbon-coated electron microscope grids for TEM characterization. The grids were subsequently washed with PBS for 3 min in lead oxalate solution at pH 7 before being imaged with an FEI Tecan 12 transmission electron microscope (FEI, Hillsboro, OR, USA).

NTA was conducted to characterize the EVs’ size and particle number using a NanoSight NS500 instrument (NanoSight, United Kingdom) equipped with a 488 nm laser module and NTA software version 3.1. A total of five 30-second videos were detained for each sample. The rate of Brownian motion of EV was measured by the NS500 instrument in a light-scattering system, with each sample being diluted 1:160 with DPBS before analysis. Polystyrene latex beads (100 nm, Malvern NTA 4088) were utilized as a positive control and PBS was employed as a negative control. The camera level was set at 14 and the detection threshold was set to five for all videos. The concentration, number of particles, mean and mode of the particle sizes were determined by processing and analyzing each video file. The SEC-EV purity was calculated by the number of EV particles per µg of protein.

### EXO-NET EVs and SEC-EVs characterization

The magnet microbeads were designed for on-bead analysis (e.g., Fourier-transform infrared spectroscopy, FTIR) or on-bead lysis and downstream analysis (e.g., ELISA, Western Blot, mass spectrometry, and NGS), thus, TEM and NTA are inappropriate methods for characterization. Following the MISEV2018 guidelines^[44]^, SEC-EVs and EXO-NET EVs were both characterized by FTIR (-3 μL), 3,3′-dioctadecyloxacarbocyanine perchlorate (DiO)-labelled EVs, CD9 ELISA (-10 μL), and bicinchoninic acid assay (BCA-2 μL) and EV surface markers by a MACSPlex Exosome Kit (10 μL).

FTIR was used to detect the EVs’ biochemical components, such as nucleic acids (PO^2-^ bonds at 1,000-1,200 cm^-1^), lipids (bonds of CH_2_ at 1,420, 2,880, and 2,920 cm^-1^), and proteins (bonds of Amide I, II, III at 1,650, 1,550, and 1,350 cm-^1^) following our previous published protocol^[35]^. The Nicolet iS20 FTIR Spectrometer (Thermo Fisher) was used in the transmission mode for FTIR analysis to explore the significant peaks in the EVs^[35,45]^. The acquisition of spectra in the range of 4,000-400 cm^-1^ at a resolution of 4 cm^-1^, along with 256 scans per sample, was facilitated by placing 2 μL of EVs on the surface of the diamond. PBS was applied as a blank and all samples were measured in triplicates.

DiO is typically employed to visualize the EVs’ morphology. Salivary EVs were incubated with a lipophilic green, fluorescent dye DiO at the final concentration of 25 μg/mL for 1 h at room temperature. Salivary EV morphology was visualized by a Leica TCS SP5 scanning laser confocal microscope.

PeproTech ELISA Development kits were used to identify the surface marker CD9 in isolated intact SEC-EVs and EXO-NET EVs, as described in a previous publication^[5]^. The 96-well plates were covered with 2 μg/mL of anti-human CD9 capture antibody (HansaBioMed, Lonza) overnight at room temperature, followed by 1-hour blocking to prevent non-specific binding. Following that, 5 μL of EV sample diluted in 45 μL of PBS was introduced to each well and cultured for 2 h at 37 ℃. After 4 times wash, 50 μL of monoclonal biotin-conjugated anti-human CD9 antibody (HansaBioMed, Lonza) at 2 μg/mL was included and covered for 2 h. After adding 50 μL of streptavidin-HRP, the plates were covered with 3,3,5,5′-tetramethylbenzidine (TMB) substrate under dark for 30 min and paused with 100 μL of 1 M HCl. The samples were measured at 450 nm using a Tecan Infinite M200 Pro spectrophotometer (Tecan).

EV protein was extracted via RIPA buffer and Pierce BCA Protein Assay Kit (ThermoFisher Scientific) was used to determine the protein concentration in EVs according to the manufacturer's instructions.

The surface marker profiles of SEC-EVs and EXO-NET EVs were characterized using the human MACSPlex Exosome Kit (Miltenyi Biotec), containing 37 EV surface markers. 10 μL EVs from three matched donors were diluted to 120 μL MACSPlex buffer and, subsequently, mixed with 15 μL of MACSPlex exosome capture beads overnight, employing a tube rotator set at 12 rpm. 5 μL labelled antibodies CD9, CD63, and CD81 were applied for counterstaining. Meticulous determination of background-corrected median fluorescence intensity (MFI) values for EVs surface marker was carried out through the subtraction of MFI values obtained from their corresponding IgG control values.

### Periodontal pathogen detection for SEC-EVs and EXO-NET EVs using genomic DNA qPCR

Genomic DNA quantitative PCR was conducted to detect periodontal or oral pathogens within enriched EVs. Total DNA was isolated from 20 µL of SEC-EVs and EXO-NET EVs using the Purelink^TM^ Microbiome DNA Purification Kit (Invitrogen, ThermoFisher Scientific). The assessment of RNA quality and quantity was conducted by a Nanodrop spectrophotometer (ThermoFisher Scientific). Real-time PCR was conducted using the SYBR Green qPCR Master Mix (ThermoFisher Scientific) on an ABI StepOnePlus instrumentation. The assay utilized primers targeting five periodontal pathogens (*Tannerella forsythia, Porphyromonas gingivalis, Treponema denticola, Peptostreptococcus anaerobius, and Eikenella corrodens*), and a 16S rRNA gene as a housekeeping gene [Supplementary Table 1]. The expression level of each DNA was normalized to the housekeeping gene and calculated as 2^-(normalized average Cts)^^[46]^.

### Inflammatory cytokines detection for EXO-NET EVs by ELISA

ELISA was used to measure inflammatory cytokines in salivary EXO-NET EVs. Lysed EV samples were subjected to ELISA to measure periodontal inflammation markers in EXO-NET EVs from both periodontitis and non-periodontitis subjects. To quantify the levels of IL-6, IL-1β, IL-8, and IL-10 inflammatory cytokines, commercially available PeproTech ELISA Development kits were utilized [Figure 1C]. The 96-well plates were coated overnight at room temperature with capture antibodies for IL-6 (0.5 μg/mL), IL-1β (0.25 μg/mL), IL-8 (0.125 μg/mL), and IL-10 (1.0 μg/mL), followed by a 1-hour blocking step to prevent non-specific binding. Subsequently, each well received 3 μL of EV sample diluted in 47 μL of PBS and incubated for 2 h at 37℃. Detection antibodies for IL-6 (0.1 μg/mL), IL-1 β (0.25 μg/mL), IL-8 (0.25 μg/mL), and IL-10 (0.50 μg/mL) diluted in PBS were then added and incubated for 2 h. After introducing 50 μL of streptavidin-HRP, the plates were incubated in the dark with 100 μL of 3,3,5,5’-tetramethylbenzidine (TMB) substrate for 30 min, and the reaction was halted with 100 μL of 1 M HCl. Absorbance was measured at 450 nm using a Tecan Infinite M200 Pro spectrophotometer.

### Statically analysis

The graph data are displayed as the median ± 95% confidence interval (CI) unless specified otherwise. Statistical differences between SEC-EVs and EXO-NET EVs, as well as between non-periodontitis and periodontitis groups, were analyzed in GraphPad Prism 9.0 software (GraphPad Software, San Diego, CA, USA). Paired t-test was used to compare between SEC-EVs and EXO-NET EVs. Unpaired t-tests with Mann-Whitney tests were employed to compare non-periodontitis and periodontitis data after a normality test by quantile-quantile plots in Prism, indicating a non-normal distribution. Statistical significance was defined as a two-tailed *P*-value < 0.05.

## RESULTS

### Demographic and clinical data for the recruited participants

A total of 32 subjects were recruited, with 12 subjects diagnosed with non-periodontitis and 20 subjects with periodontitis, as indicated in Table 1. The cohort consisted of 11 females (34.37%) and 21 males (65.63%), with ages ranging from 23 to 87 years. Remarkably, a statistically significant age difference was observed between the periodontitis and non-periodontitis groups (*P* < 0.0001). The ethnic backgrounds of the 32 subjects were diverse, primarily comprised of Caucasian and Asian individuals. In the periodontitis group, both BOP % and PI % values were significantly higher compared to that in the non-periodontitis group (*P* < 0.0001). Furthermore, among the 20 patients diagnosed with periodontitis with an average PPD of 4.85 mm and 28 sites ≥ 5mm, 11 patients (55%) had stage III periodontitis, 9 (45%) patients had stage IV periodontitis, 14 patients (70%) had grade B periodontitis, and 6 patients (30%) had stage C periodontitis.

**Table 1:** Demographic data and clinical data for participants

		**Non-periodontitis** **(*n* = 12)**	**Periodontitis** **(*n* = 20)**
Gender	Male	8 (66.67%)	13 (65.00%)
	Female	4 (33.33%)	7 (35.00%)
Age		33.58 ± 7.34 (23-48)	58.95 ± 12.99 (37-87) *P* < 0.0001
Ethnicity	Caucasian	8 (66.67%)	17 (85%)
	Asian	4 (33.3%)	3 (15%)
BOP (%)		29.08% ± 0.19 (6%-70%)	36.54% ± 0.15 (9%-83%) *P* < 0.0001
PI (%)		34.83% ± 0.27 (6%-89%)	41.33% ± 0.24 (15%-90%) *P* < 0.0001
No. of sites (≥ 5mm)			28.75 ± 17.91 (5-66)
Average PPD (mm)		-	4.85 ± 1.41 (2.89-8.00)
Periodontitis classification	Grade B	-	14 (70%)
	Grade C	-	6 (30%)
	Stage III	-	11 (55%)
	Stage IV	-	9 (45%)

*P* values *vs*. non-periodontitis using the Mann-Whitney test. Data are displayed as mean ± standard deviations. No. of sites > 5 mm: the number of specific locations around teeth where the PPD is measured and found to be greater than 5 mm. BOP: bleeding on probing, the presence of bleeding from the gingival sulcus or periodontal pocket when a periodontal probe is gently inserted into this space; PI: plaque index, a measure of the amount of dental plaque on tooth surfaces; PPD: probing pocket depth, the measurement of the depth of the gingival sulcus or periodontal pocket.

### SEC-EVs characterization

**Figure 2:**
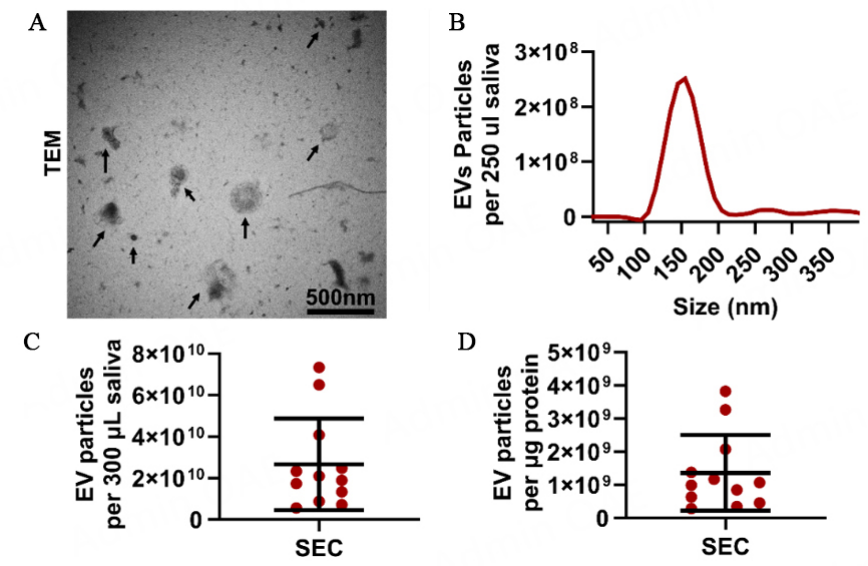
SEC-EVs characterization. (A) TEM analysis (arrows denote SEC-EVs); (B) particle size histogram distribution after NTA analysis; (C) Salivary EV particle number per 300 µL saliva; (D) EV purity was defined as EV particle number per µg protein. TEM: transmission electron microscopy.

Salivary EVs were isolated from 12 non-periodontitis participants using the SEC method and EXO-NET as described in Figure 1A and B. It is noted that SEC-EVs were characterized using TEM and NTA [Figure 2]; however, TEM and direct NTA methods are not suitable for characterizing EVs bound to paramagnetic beads for EXO-NET EVs.

To confirm the presence of EVs and determine their size and morphology, TEM and NTA were employed for SEC-EVs. TEM analysis revealed the presence of circular vesicles ranging from 30 nm to 200 nm in diameter [Figure 2A]. Additionally, NTA results indicated the total number of EV particles in 300 µL saliva was 2.66 × 10^10^ particles and the SEC-EVs purity was 1.36 × 10^9^ particles per µg protein [Figure 2C and D]. It is noted that NTA can only provide particle number and size information but is not capable of differentiating EVs from similar-sized non-EV particles.

### SEC-EVs and EXO-NET EVs characterization

**Figure 3:**
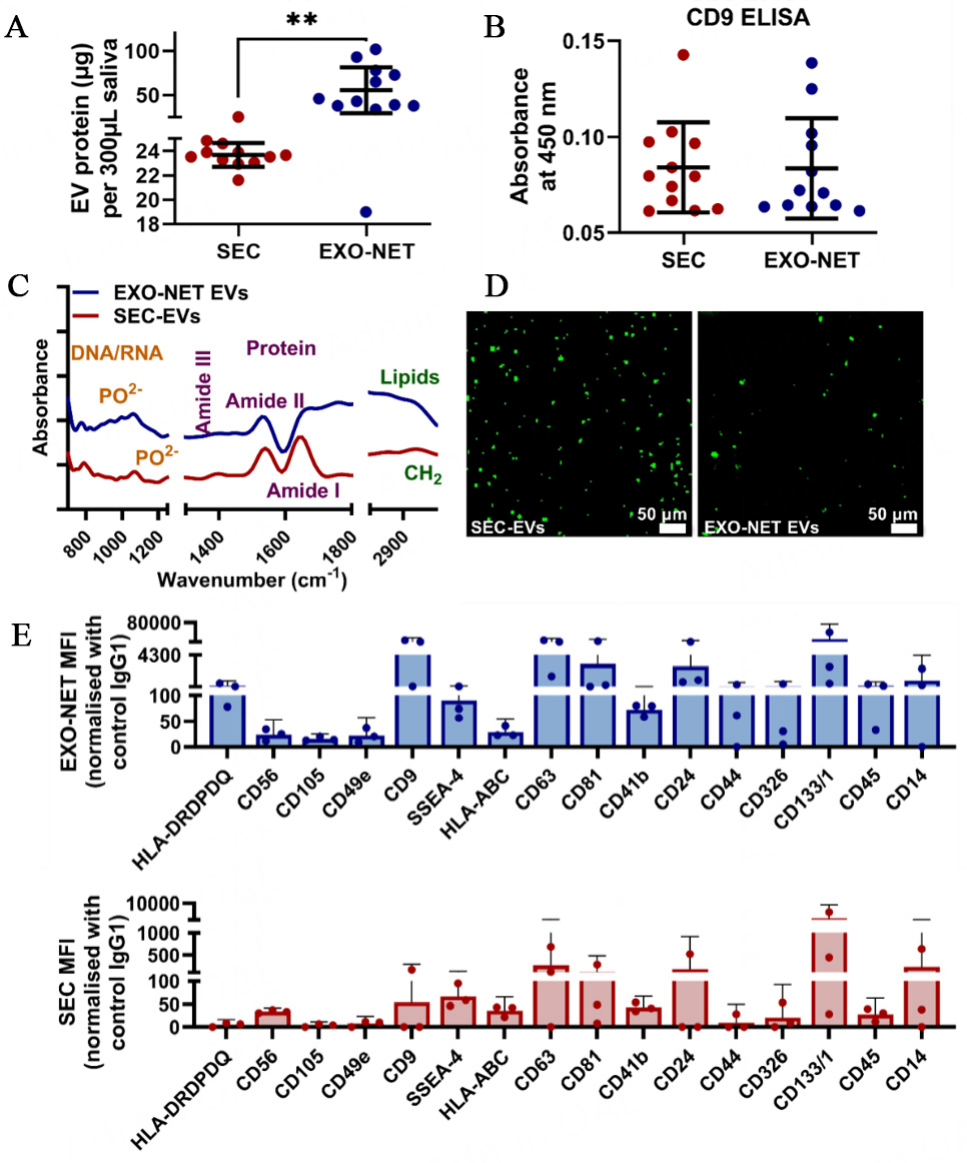
Characterization of SEC-EVs and EXO-NET-enriched EVs included: (A) Quantification of protein content using the BCA assay; (B) Identification of CD9+ EV subpopulation; (C) Analysis of EVs spectra using FTIR; (D) Visualization of DiO-labeled EVs; (E) Selective EV surface marker profiles for SEC-EVs and EXO-NET EVs using a multiplex Exosome kit. Three dots indicate data from three donors. **P < 0.01 between SEC-EVs and EXO-NET EVs groups.

For both SEC-enriched EVs (SEC-EVs) and EXO-NET-enriched EVs, EVs characterization assessed protein content, CD9 ELISA, FTIR, and DIO-EV morphology and 37 EV surface markers with a multiplex exosome kit in Figure 3. Protein analysis by BCA assay showed that EXO-NET EVs contained 2-3 times more protein content compared to SEC-EVs [Figure 3A]. CD9 expression levels in salivary EVs were also evaluated, and CD9 ELISA results showed no significant difference between 10µL of SEC-EVs and EXO-NET EVs [Figure 3B]. Further characterization of EVs using FTIR showed the detection of DNA/RNA with PO^2-^ bonds at 1,000-1,200 cm^-1^, protein of Amide I, II, III at 1650, 1550, and 1350 cm^-1^, and lipid of CH_2_ peak around 1,420, 2,880, and 2,920 cm^-1^ [Figure 3C]. Finally, DiO-tracking provided visualization of the distribution of EVs, revealing that EXO-NET EVs exhibited a larger size compared to SEC-EVs due to the inclusion of microbeads within the EXO-NET kit [Figure 3D].

We further performed a comprehensive analysis of EV surface markers on both SEC-EVs and EXO-NET EVs, as illustrated in Figure 3E. Our research findings revealed that EXO-NET EVs showed higher expression for all investigated EV surface markers compared to that from SEC-EVs. Of note, the variations in the higher expression levels of CD9, CD63, and CD81 within salivary EVs were observed in EXO-NET EVs compared to SEC-EVs. This indicates that EXO-NET is a potential superior EV enrichment method to enrich host-derived EVs compared to the SEC method.

The data presented in Figure 3 demonstrated the successful characterization of EVs using both SEC and EXO-NET methods, following the latest MISEV2018 guidelines.

### Significantly reduced bacterial DNA detection in salivary EXO-NET EVs

**Figure 4:**
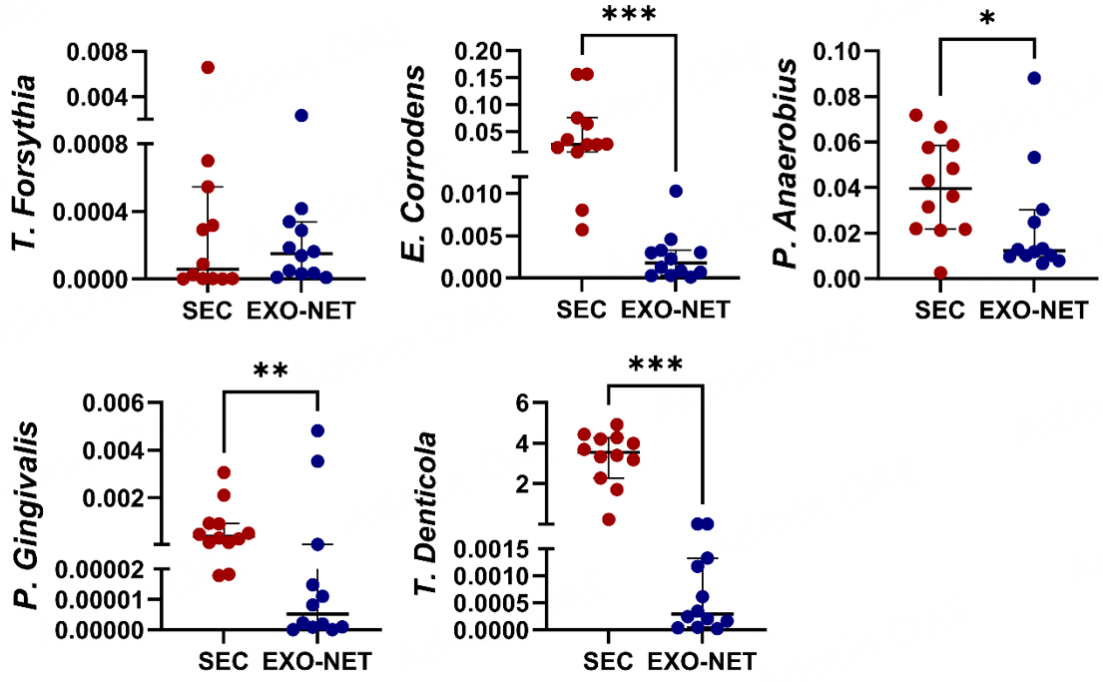
The expression of bacterial genes in SEC-EV and EXO-NET-enriched EVs was evaluated. The bacterial genes demonstrated a downward trend in EXO-NET EVs compared to SEC-EVs. The results were presented as a scatter dot plot graph, displaying the median values with a 95%CI. *P < 0 .05, **P < 0 .01, ***P < 0 .001, between SEC-EVs and EXO-NET EVs groups.

To evaluate the impact of SEC and EXO-NET methods on bacteria expression of salivary EVs from non-periodontitis (*n* = 12) individuals, we performed bacterial DNA expression of SEC-EVs and EXO-NET EVs followed by genomic DNA PCR. The results showed that except for *T. Forsythia* [Figure 4], other bacterial DNA for *E. corrodens, P. anaerobius, P. gingivalis*, and *T. denticola* [Figure 4] had statistically significant differences between SEC-EV and EXO-NET EV. Notably, EXO-NET EVs consistently exhibited much lower expression levels of all five bacterial genes and contained lower levels of periodontal pathogens compared to SEC-EVs. This further demonstrated that EXO-NET can potentially reduce bacterial EVs or bacteria DNA contamination compared to the SEC method.

### Cytokine levels in EXO-NET enriched salivary EVs for periodontitis

**Figure 5:**
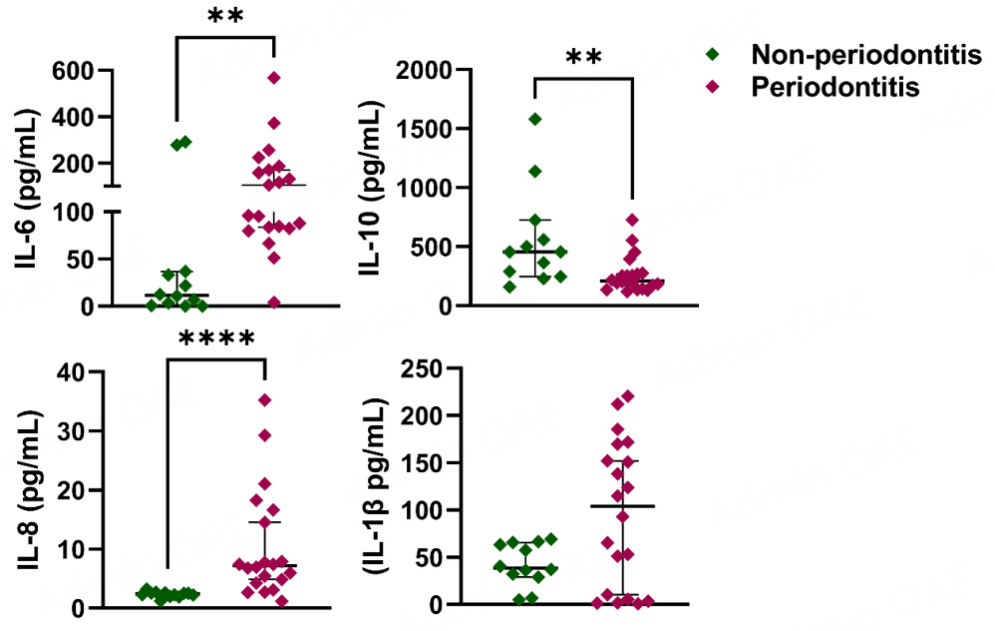
The differential expression of cytokines in EXO-NET EVs in both non-periodontitis and periodontitis groups. Significantly elevated IL-6 and IL-8 and reduced IL-10 were observed in the periodontitis salivary EVs compared to the non-periodontitis group. The results were visualized using scatter dot plot graphs, presenting the median values with a 95% CI. Significance levels were indicated as * *P* < 0.05 and ** *P* < 0.01, **** *P* < 0 .0001, indicating differences between the periodontitis and non-periodontitis groups.

The expression levels of four inflammatory cytokines, IL-6, IL-10, IL-8, and IL-1β, were evaluated in salivary EXO-NET EVs of non-periodontitis and periodontitis patients using ELISA [Figure 5]. The investigation showed significant differences in three cytokines, IL-6 (*P* < 0.01), IL-10 (*P* < 0.01), and IL-8 (*P* < 0.0001), between non-periodontitis and periodontitis patients. IL-6 and IL-8 were significantly upregulated, and IL-10 was downregulated in the periodontitis group compared to the non-periodontitis group. No statistically significant difference in IL-1β was identified.

## DISCUSSION

The data obtained in this study establish that immunoaffinity-bead capture (EXO-NET) is a reliable technique for enriching salivary EVs with significantly lower bacterial DNA content than the SEC method. EXO-NET EVs display increased IL-6, IL-8, and IL-1β in periodontitis individuals compared to those without, with significantly reduced IL-10 expression. These findings support considering salivary EVs as a potential alternative diagnostic tool for periodontitis.

Saliva is a non-invasive and easily accessible method for detecting oral diseases, and EVs found in saliva and blood have the potential to serve as periodontitis biomarkers^[15]^. EVs have a stable lipid bilayer structure and are abundant in biological fluids such as saliva and blood, enabling them to be a potential source of periodontitis biomarkers^[26,27]^. However, the isolation of salivary EVs faces significant challenges, and current methods for isolating EVs from biological fluids include SEC, bead-based immunoaffinity capture, and ultracentrifugation (UC)^[26-28,47]^. Available data from Wang *et al*., Iliuk *et al*., Chen *et al*., and Mussack *et al*. prove that magnetic bead-based immunoaffinity capture is a more efficient and effective method for EV enrichment from plasma or urine than UC or SEC, with a higher yield and purity^[47-51]^. Our experimental results indicate that the protein concentration of EXO-NET EVs was almost two to three times higher than that of SEC-EVs, suggesting that EXO-NET EVs may have a higher yield with less microbial DNA detection compared to SEC, consistent with published data^[47,48]^. The CD9 ELISA data indicate that immunoreactive CD9 is present in both the SEC and EXO-NET preparations. Furthermore, multiplex EVs assay results showed that the surface marker expression in EXO-NET EVs was significantly higher than in SEC-EVs.

Through the data obtained from bacteria genomic DNA Qpcr [Figure 4], it is worth noting that EXO-NET EVs have significantly less periodontal pathogenic DNA expression, indicating EXO-NET led to more host-enriched EVs by reducing microbial EV components. It appears that the EXO-NET technique could potentially be a superior approach for eliminating bacterial impurities in EVs derived from saliva, resulting in enhanced purity. Conversely, the SEC method may concentrate EVs from both the host and bacteria, thus reducing overall purity. We speculate that eliminating microbial EVs from salivary EV populations can potentially enhance the precision and sensitivity of saliva-based diagnostics, particularly in assessing host inflammatory responses. Our proof-of-concept data illustrate that immunoaffinity methods provide a robust and specific approach for isolating and detecting host-derived EVs in saliva, which may achieve high diagnostic specificity and sensitivity. Nevertheless, additional research is necessary to validate the effectiveness of this method in the context of EV diagnosis, particularly in larger sample sizes.

Periodontitis is a prevalent chronic disease that triggers the production of proinflammatory cytokines such as IL-6, IL-8, and IL-1β as a response to bacterial infection^[52]^. Conversely, IL-10 is an anti-inflammatory cytokine that plays a regulatory role in dampening the immune response and preventing excessive inflammation. Salivary EVs are a rich source of various biomolecules, including cytokines, which can provide insights into the physiological and pathological processes in the body^[52]^. In the case of periodontitis, cytokines are considered to play a crucial role in the disease’s pathogenesis. Acharya *et al.* showed a positive correlation between IL-6 and BOP%, suggesting a strong proinflammatory effect that enhances alveolar bone destruction and affects vascular homeostasis^[53]^. Studies by Enver *et al*., Gündoğar *et al.*, and Al-Hamoudi *et al.* have also revealed that IL-1β levels increase and IL-10 levels decrease in the presence of periodontitis, leading to dysregulation of the expression of inflammatory cytokines^[54-56]^. Hoare *et al.* and Bakhsh *et al.* have reported an increase in the mRNA levels of *IL-1β, IL-6, and IL-8* in gingival tissue samples^[57,58]^. Several papers reveal that salivary IL-6, IL-8, IL-10, and IL-1β could be potential markers for periodontitis^[38-42]^. Our study found that periodontitis EXO-NET EVs exhibited increased levels of IL-6, IL-8, and IL-1β compared to non-periodontitis EVs, which is consistent with published levels of these cytokines in saliva^[38-42]^ and gingival tissues^[54-56]^ in periodontitis. The observed downregulation of IL-10 in the salivary EVs derived from periodontitis suggests a protective response in healthy individuals, aiming to prevent disease^[59]^. Compared with the salivary cytokine concentrations reported in other studies^[60-62]^, we observed that the Interleukin concentration in our experiment was higher than that reported in studies exploring cytokine expression in periodontitis using saliva samples. This suggests that EVs may potentially offer greater sensitivity in detecting cytokine expression. However, further investigation is required to confirm this observation. These findings indicate that accurately detecting and quantifying these cytokines in salivary EVs could provide a non-invasive way to diagnose and monitor periodontitis.

One of the study's limitations includes a relatively small patient sample size and age variation between non-periodontitis participants and those with periodontitis (*P* < 0.0001). The age difference may have affected the components of salivary EVs^[63,64]^. Additionally, we did not distinguish between healthy and gingivitis samples, which may also be the reason why there was no significant difference in IL-β levels in cytokine expression between the non-periodontitis and periodontitis groups. An additional limitation in this study is the absence of data on saliva flow rate, and the exclusion of periodontitis patients in Aim 1, where we aimed to investigate two methods for enriching salivary EVs: SEC, and EXO-NET beads. Regarding the characterization of EVs, there was no western blot to evaluate cytosolic EV protein markers. Consequently, future studies involving larger cohorts with age-matched individuals in both healthy and periodontitis groups are warranted. Despite these limitations, cytokine levels in salivary EVs can provide valuable information about the inflammatory response and immune status in periodontitis patients. Measuring these cytokines could serve as potential diagnostic biomarkers for periodontitis.

This study represents a considerable advancement in the field of periodontitis diagnosis research and highlights the potential of EVs as a source of disease biomarkers. Additional study is required to validate these findings and explore the clinical applicability of EV-based biomarkers. Nonetheless, the results provide a promising foundation for future investigations into the use of EVs as diagnostic and therapeutic tools in the fight against periodontitis.

## CONCLUSIONS

This study demonstrates the reliability of immunoaffinity capture as a method for enriching host-derived salivary EVs with less detection of bacterial DNA. The findings suggest that immunoaffinity-enriched EVs could serve as potential biomarkers for periodontitis, as evidenced by increased expression of proinflammatory cytokines observed in EVs from periodontitis patients. Moreover, the characterization of cytokine expression patterns in EVs from periodontitis patients could enhance our understanding of the disease's pathogenesis and aid in the development of targeted therapeutic interventions.

## DECLARATIONS

### Availability of data and materials

Data will be available upon request.

### Copyright

© The Author(s) 2023.
