# Supplementary material for: Immunoaffinity-enriched salivary small extracellular vesicles in periodontitis
Source: Extracell Vesicles Circ Nucl Acids. 2023 Dec 30;4(4):698–712. doi: 10.20517/evcna.2023.48 (PMC11648426; doi:10.20517/evcna.2023.48)
Supplement: Supplementary file 1 [file evcna-4-4-698-SupplementaryMaterials.pdf]

Supplementary Material

Immunoaffinity-enriched salivary small extracellular vesicles in periodontitis

Chun Liu[1], Chaminda Jayampath Seneviratne[1], Carlos Palma[2], Greg Rice[2,3], Carlos Salomon[3], Ramin Khanabdali[2], Sašo Ivanovski[1,*], Pingping Han[1,*]

[1]Epigenetics nanodiagnostic and therapeutic group, Center for Oral-facial Regeneration, Rehabilitation and Reconstruction (COR3), School of Dentistry, The University of Queensland, Brisbane, QLD 4006, Australia.
[2]INOVIQ Limited, Notting Hill, VIC 3168, Australia.
[3]Translational Extracellular Vesicles in Obstetrics and Gynae-Oncology Group, University of Queensland Centre for Clinical Research, Faculty of Medicine, Royal Brisbane and Women's Hospital, The University of Queensland, Brisbane, QLD 4029, Australia.

Correspondence to: Dr. Pingping Han, Epigenetics nanodiagnostic and therapeutic group, Rehabilitation and Reconstruction (COR3), Center for Oral-facial Regeneration, School of Dentistry, The University of Queensland, 288 Herston Road, Brisbane, QLD 4006, Australia. E-mail: p.han@uq.edu.au; Prof. Sašo Ivanovski, Rehabilitation and Reconstruction (COR3), Center for Oral-facial Regeneration, School of Dentistry, The University of Queensland, 288 Herston Road, Brisbane, 4006, Australia. E-mail: s.ivanovski@uq.edu.au

Supplementary Table 1. Primers for periodontal pathogens

Pathogen.	Forward primer (5'-3')	Reverse primer (5'-3')
Porphyromonas gingivalis	TGGTTTCATGCAGCTTCTTT	TCGGCACCTTCGTAATTCTT
Tannerella forsythia	GGGTGAGTAACGCGTATGTAACCT	CCCATCCGCAACCAATAAA
Treponema denticola	TGGTGAGTAACGCGTGGGTGACCT	TTCACCCTCTCAGGCCGGA
Peptostreptococcus anaerobius	GGGTGAGTAACGCGTGGGT	TACTGATCGTCGCCTTGGTGG
Eikenella corrodens	ACGTCCTACGGGAGAAAGCGG	CCATTGTCCAAAATTCCCCACTG
16S rRNA	CGGCAACGAGCGCAACCC	CCATTGTAGCACGTGTAGCC
